# A versatile dataset for intrinsic plagiarism detection, text reuse analysis, and author clustering in Urdu

**DOI:** 10.1016/j.dib.2023.109857

**Published:** 2023-11-26

**Authors:** Muhammad Haseeb, Muhammad Faraz Manzoor, Muhammad Shoaib Farooq, Uzma Farooq, Adnan Abid

**Affiliations:** a Department of Computer Science, University of Management and Technology, Lahore, Pakistan; b Department of Data Science, Faculty of Computing and Information Technology, University of the Punjab, Pakistan

**Keywords:** Plagiarism detection, Intrinsic plagiarism, Stylometry features, Sentence, Paragraph, Urdu language

## Abstract

Plagiarism detection (PD) is a process of identifying instances where someone has presented another person's work or ideas as their own. Plagiarism detection is categorized into two types (i) Intrinsic plagiarism detection primarily concerns the assessment of authorship consistency within a single document, aiming to identify instances where portions of the text may have been copied or paraphrased from elsewhere within the same document. Author clustering, closely related to intrinsic plagiarism detection, involves grouping documents based on their stylistic and linguistic characteristics to identify common authors or sources within a given dataset. On the other hand, (ii) extrinsic plagiarism detection delves into the comparative analysis of a suspicious document against a set of external source documents, seeking instances of shared phrases, sentences, or paragraphs between them, which is often referred to as text reuse or verbatim copying. Detection of plagiarism from documents is a long-established task in the area of NLP with remarkable contributions in multiple applications. A lot of research has already been conducted in the English and other foreign languages but Urdu language needs a lot of attention especially in intrinsic plagiarism detection domain. The major reason is that Urdu is a low resource language and unfortunately there is no high-quality benchmark corpus available for intrinsic plagiarism detection in Urdu language. This study presents a high-quality benchmark Corpus comprising 10,872 documents. The corpus is structured into two granularity levels: sentence level and paragraph level. This dataset serves multifaceted purposes, facilitating intrinsic plagiarism detection, verbatim text reuse identification, and author clustering in the Urdu language. Also, it holds significance for natural language processing researchers and practitioners as it facilitates the development of specialized plagiarism detection models tailored to the Urdu language. These models can play a vital role in education and publishing by improving the accuracy of plagiarism detection, effectively addressing a gap and enhancing the overall ability to identify copied content in Urdu writing.

Specification Table

**Table:** 

Subject	Computer Science
Specific Subject Area	Machine Learning, Deep Learning
Data Format	Raw
Type of Data	Intrinsic Plagiarism Documents, Extrinsic Plagiarism Documents, Author Clustering, Text Reuse
Data Collection	The documents are extracted from the Urdu based websites. Documents are based on the most discussed topics in Urdu language such as Quaid-e-Azam, Independence Day etc. The basic text statistics including total documents, number of plagiarized documents, number of unplagiarized documents, minimum, maximum and average length of documents were obtained through text complexity analysis.
Data source location	Details are provided in Appendix A.
Data accessibility	https://data.mendeley.com/datasets/rbpm4pw24r/3

## 1 Value of the Data


 

• This dataset serves as a pioneering resource for a variety of critical tasks within the Urdu language.
• Intrinsic Plagiarism Detection: Facilitating the development and evaluation of models tailored for detecting plagiarism within the same document.
• Author Clustering: Supporting the grouping of documents based on linguistic and stylistic attributes to identify common authors or sources.
• Text Reuse (Verbatim) Detection: Enabling the identification of shared phrases, sentences, or paragraphs between documents.
• It holds the distinction of being the first multipurpose corpus tailored specifically for the Urdu language, offering researchers and practitioners a versatile tool for diverse applications in natural language processing and text analysis.
• It aids in developing language-specific attributes, leveraging unique distinctions to bolster detection precision.

## 2 Data Description

**Fig. 1:**
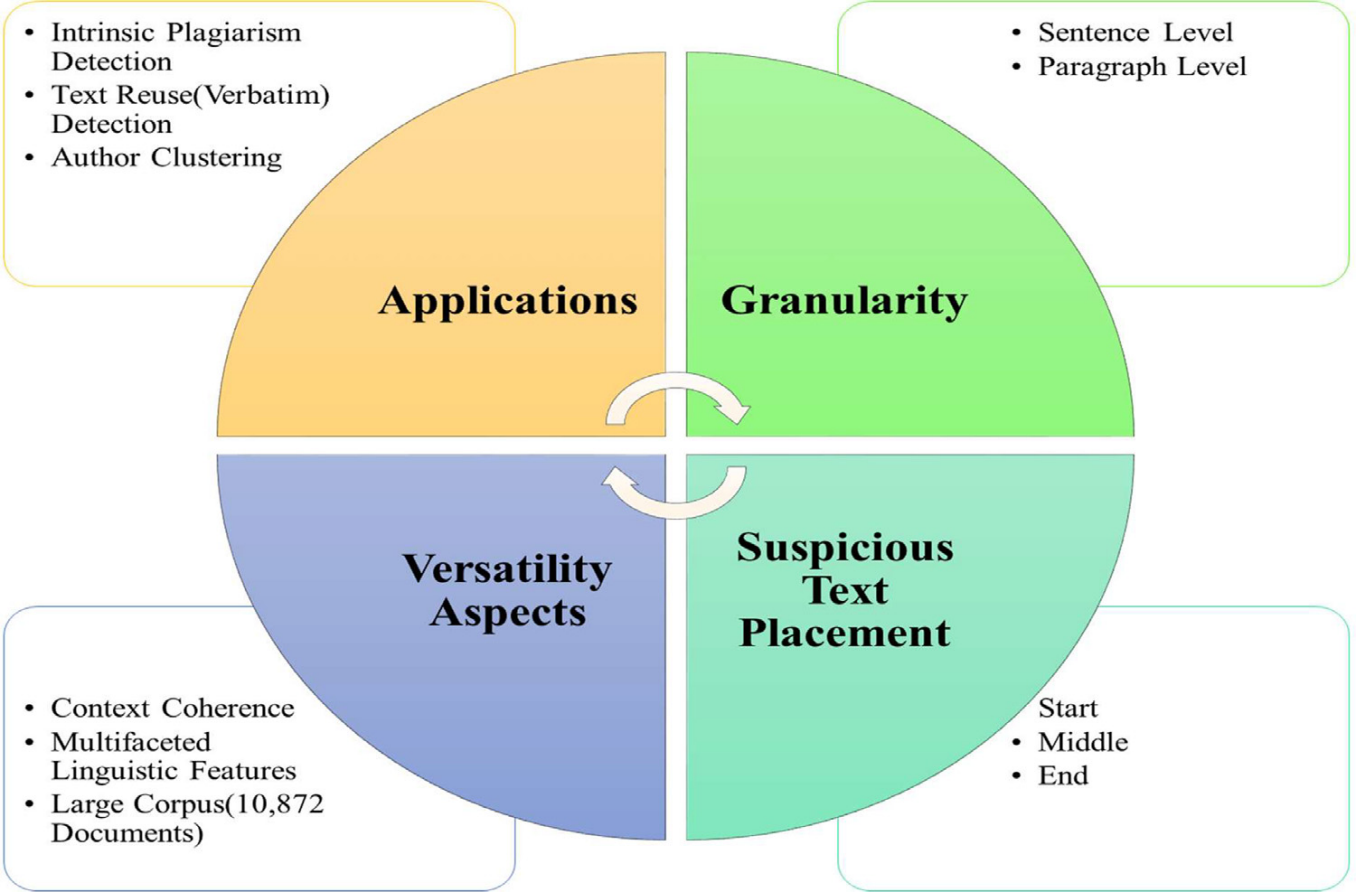
Salient features of the proposed corpus.

Urdu is very famous language in South Asian region as more than 85 million people speak Urdu [1]. However, being a low-resource language, no appropriate, relevant, and high quality corpus for plagiarism detection in Urdu exist. Hence, no intelligent plagiarism detection models for Urdu language have been developed so far. To the best of our knowledge, this is the first systematic effort to build useful corpus for various plagiarism detection related tasks in Urdu language. In this paper, a multipurpose corpus for various types of plagiarism detection tasks is systematically developed. The salient features of the proposed corpus are shown in Fig. 1. To produce a high-quality corpus, we have gathered the Urdu essays and reports from various popular and highly trending websites such as, jang.com, urduessaypoint.blogspot.com, www.dawnnews.tv etc. The categorization details for these topics are outlined in Table 1.

**Table 1:** Topics and essay mapping.

Notably the dataset comprises a diverse array of documents, encompassing various genres prevalent in Urdu writing. It includes national celebrities, annual events and moral lessons of textual forms, ensuring a representative compilation of Urdu language content. This composition allows for a comprehensive exploration of the intricacies of plagiarism detection across different types of documents.

On the other hand, each of the document is further divided in range of well-defined topics, covering a wide range of subjects and domains within the Urdu language. This thematic richness ensures that the dataset is applicable to a multitude of scenarios and caters to the diverse writing landscape in Urdu. Moreover, the dataset meticulously captures the multifaceted landscape of writing styles in Urdu, incorporating a plethora of stylometric features. From lexical choices to sentence structures, the dataset includes diverse linguistic nuances, allowing for an in-depth exploration of writing styles. Stylometric features such as average word length, sentence length, and the ratio of long words contribute to the comprehensive representation of how authors express themselves uniquely. Additionally, the dataset considers the frequency of punctuation marks, providing insights into the punctuation preferences of different writers. The final corpus comprises 10,872 documents, strategically divided into both single and paragraph granularity levels, enhancing its versatility and usability. Overall characteristics are shown in Table 2.

**Table 2:** Overall characteristics of corpus.

Characteristics	Document Type	
	Plagiarized	Non-Plagiarized
Number of Documents	5436	5436
Maximum Length of Document (in words)	7205	5285
Minimum Length of Document (in words)	129	373
Total no of words in all files	7994945	7061590
Total number of unique words in all files	18047	120913
Average number of characters in a word	3.64	3.62
Average Sentence Count	55.03	42.23
Average word count(per document)	1469.12	1297.41

The compilation process spanned approximately 9 months, where 5436 documents are plagiarized and 5436 documents are non-plagiarized according to the well-defined rules and guidelines. The corpus is made available in both .txt and .xlsx formats, providing diverse access options. Granularity wise comprehensive insights regarding the constructed corpus are listed in Table 3.

**Table 3:** Granularity wise characteristics of corpus.

Characteristics	Granularity	
	Para Level	Sentences Level
Number of Documents	2718	2718
Maximum Length of Document (in words)	7205	6819
Minimum Length of Document (in words)	253	129
total no of words in all files	4173294	3821651
total number of unique words in all files	16304	17389
average number of characters in a word	3.64	3.64
Average Sentence Count	58.07	51.99
Average word count	1533.75	1404.49

## 3 Experimental Design, Materials and Methods

**Fig. 2:**
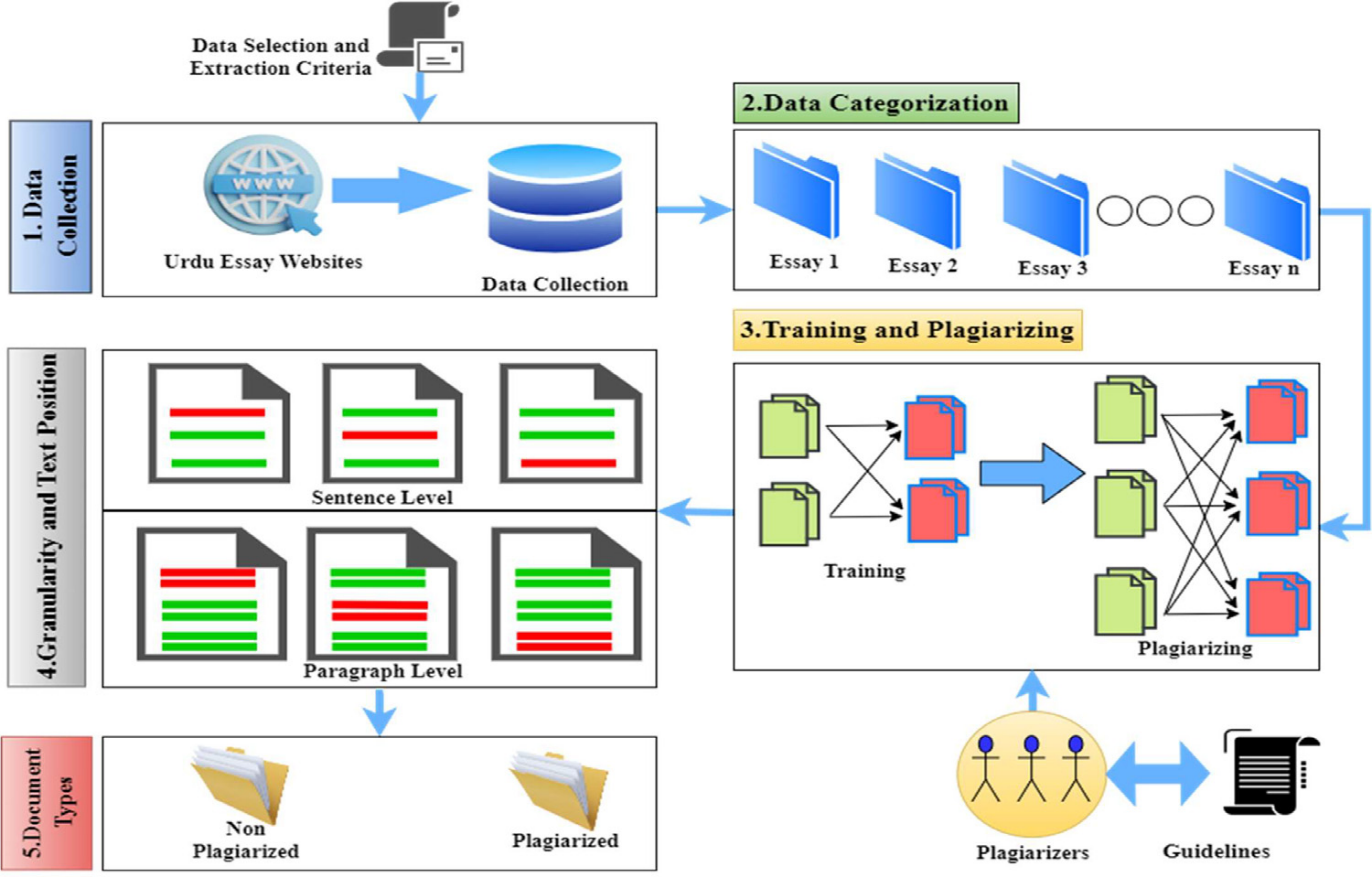
Corpus generation process.

The primary objective for developing this corpus to facilitate the researchers to assess evaluate the state of the art plagiarism detection systems. A structured methodology has been established to create and ensure the excellence of the proposed corpus as shown in Fig. 2. The methodology encompasses: (i) *Establishing Data collection criteria:* This stage includes the set of guidelines that need to be followed when extracting the data (ii) *data collection*: in this step, essays are extracted from prominent Urdu websites, forming the benchmark corpus. These essays are then organized based on topic similarities. (iii) *Process of Plagiarizing the Documents*: During this phase, documents are methodically emulated with plagiarism, adhering to meticulously outlined guidelines. (iv) *Granularity of the Plagiarized Documents:* This stage includes the systematic segregation of the corpus in sentence and paragraph level document.

### 3.1 Data Collection Criteria

We set different criterion that should be verified in the target documents as shown in Table 4 below: (documents where plagiarized fragments will be inserted).

**Table 4:** Data selection and extraction criteria.

Criteria#	Criteria Name	Description
C1	Single-Origin Document	Each target document must be extracted from single source. Otherwise, the document will contain many writing styles which may complicate the plagiarism detection even further
C2	Optimal Target Document Length	Target documents should not be too short. It is because the stylistic analysis becomes unreliable with short Urdu texts as it is with short English text [2] .
C3	Punctuation Impact	Texts require proper punctuation as they undergo crucial style analysis, where punctuation holds significance. While this criterion appeared evident, it's worth noting that during later stages of text compilation, we observed subpar quality in some online Urdu texts. Consequently, numerous collected documents were excluded due to inadequate punctuation and sectioning.
C4	Copyright-Conscious Corpus Compilation Approach	To ensure a copyright-free corpus for public availability, we opted for a selective approach. We gathered texts exclusively from websites featuring topic and author tags, significantly limiting our source options. Additionally, we included texts from verified copyright-free sources.

### 3.2 Data Collection

In this step, we identified the frequently discussed essays and topics at both school and college levels. This approach was adopted to curate a corpus containing authentic examples. These topics predominantly revolved around annual national events like Independence Day and renowned personalities. Our rationale for selecting these themes was the availability of a substantial number of independently crafted documents for each topic online. To make developed corpus more versatile, we organized these essays into categories based on thematic similarities, as illustrated in Table 1(Section 1).

### 3.3 Process of Plagiarizing the Documents

To ensure high quality, we employed university-level students to undertake the document plagiarizing process. Three students (A, B, and C) were tasked for plagiarizing the documents. These students possess a robust background in natural language processing, demonstrating a thorough comprehension of plagiarism, its nuances, and related details. As native Urdu speakers, their fluency in the language further contributes to the excellence of their work.

#### 3.3.1 Suspicious Text Placement Guidelines

In the manual selection of sentence and paragraph, and their integration into the destination document, the pivotal aim is to uphold the context in which a sentence or paragraph is utilized. Clear guidelines for placing suspicious sentences and paragraph were provided to the students during the manual document plagiarizing process.

These guidelines are outlined below:

i. A suspicious text extracted from essay 2 will be inserted at the beginning of essay 1, creating a single plagiarized document.
ii. Subsequently, another suspicious text from essay 2 will be chosen and placed in the middle of essay 1, generating another plagiarized document.
iii. Similarly, a single suspicious text from essay 2 will be selected and appended to the end of essay 1, producing yet another plagiarized document. It is pertinent to mention that in the context of this study, beginning of the essay refers to the placement of suspicious text within the first paragraph, strategically integrated to seamlessly blend with the original content without disrupting the overall context. Middle of the essay pertains to the insertion of suspicious text after the first but before the last paragraph, ensuring that it harmoniously fits within the essay's narrative while maintaining coherence. Lastly, end of the essay signifies the placement of suspicious text within the last paragraph, thoughtfully positioned to align with the essay's context and flow without causing any disruptions.

In the next round, the source will change and suspicious text will be picked from next essay 3 while the destination essay will remain the same essay 01, so on essay 01 will plagiarize with all remaining essays of the same topic. In the next turn, essay 2 will plagiarize by remaining 9 essays by using the same procedure. The remaining topics will also follow the same procedure.

#### 3.3.2 Phases of Plagiarizing the Documents

The process to plagiarize the documents comprises of following three phases:

• Phase I: Training and initial plagiarism

To start, Student A and Student B underwent a training phase where they manually emulated 100 documents. They followed structured guideline for placing sentences, and later, a thorough discussion addressed any issues or discrepancies that arose.

• Phase II: Sentence integration

Following training, Students A and B collectively evaluated the remaining documents. This phase involved them classifying documents as small, medium, or large.

• Phase III: Resolving disagreements

The primary source of disagreement between the two students stemmed from pinpointing the correct location for suspicious text, which disrupted the natural progression of the target document. As part of the conflict resolution process, the guidelines for plagiarism were also updated through discussions and consensus among the plagiarizers. The disagreement on any of the document was resolved by the third student. The determination made by Student C was the ultimate decision, settling any conflicts surrounding the disputed texts' positioning.

Cohen's Kappa Statistic is utilised in this study to assess the degree of agreement among plagiarizers. Using the following formula, Cohen's Kappa score of 0.89 was obtained among plagiarizers:

(i) $$k\;=\;\left(p{\;}_{o}-\;p_{e}\right)\;/\;\left(1\;-\;p_{e}\right)$$

where:

$p_{o}$: Relative observed agreement among raters

$p_{e}$: Hypothetical probability of chance agreement

#### 3.3.3 Document Types

• Plagiarized texts: The proposed corpus encompasses two document categories: plagiarized and non-plagiarized. Within the plagiarized documents, there exists intentional variability in text length. This strategic diversity enhances corpus versatility, mirroring real-world variations in plagiarized text length. Training models with such varied content equips them to adeptly handle diverse scenarios. The scope of plagiarized text spans varying lengths within the Urdu intrinsic plagiarism detection corpus. This purposeful variation intends to heighten the corpus's adaptability. As real-world instances exhibit varying lengths of plagiarized text, training models on this versatile corpus enhances their robustness and applicability across contexts.
• Non-plagiarized documents: Noteworthy Urdu platforms like Urdu Notes, Urdu Wikipedia contain essays and topics in the Urdu language. These texts are distinct, originating from diverse authors. This approach stems from the objective to capture essays on similar themes but authored by different individuals.

#### 3.3.4 Context and Placement of Suspicious Text

Throughout the document plagiarism process, we insert suspicious text within the documents, making sure that the context remains coherent. Our manual approach to text placement strategically positions suspicious content at three distinct locations within the document: the beginning, the middle, and the end. This method of placement aims to mimic potential instances of plagiarism, where copied content may be integrated seamlessly into various parts of the text. By maintaining contextual relevance, we aim to enhance the authenticity of the detection process, making it more aligned with real-world instances of text appropriation.

### 3.4 Granularity of Plagiarized Documents

Two granularity levels are followed in this corpus to plagiarized the documents: (i) Sentence Level and (ii) Paragraph Level.

• Sentence level:

In the Sentence Level granularity, one sentence is chosen from the source document. This sentence is then positioned in three different locations (beginning, middle, and end) within the destination document. This process of placement, one after the other, constitutes the act of plagiarism. The detailed example of sentence level plagiarism is shown in Appendix B:

• Paragraph level:

Similar to the sentence level granularity explained above, the Paragraph level operates with the same procedure. The distinction lies in the content being placed within paragraphs instead of sentences. A Comprehensive example of paragraph level plagiarism is shown Appendix C.

## 4 Applications and Use of the Corpus

This multifaceted corpus extends its utility to intrinsic plagiarism detection, text reuse identification, and author clustering, offering a versatile toolset for researchers and practitioners in the realm of natural language processing and text analysis.

### 4.1 Intrinsic Plagiarism Detection

This corpus serves as a vital resource for intrinsic plagiarism detection in the Urdu language. Its versatility lies in its ability to accommodate different granularity levels, including single sentence and single paragraph. This diversity ensures that the models developed using this corpus can effectively detect instances of plagiarism regardless of the scale, making it suitable for various applications within intrinsic plagiarism detection.

• Versatile linguistic features: The incorporation of both sentence and paragraph granularity levels allows for the assessment of linguistic features in varied contexts, enriching the analysis and detection of intrinsically plagiarized content.
• Context coherence: The meticulous placement of suspicious text within documents maintains contextual coherence, enabling more accurate detection and attribution.

### 4.2 Text Reuse (Verbatim)

The corpus also finds applicability in detecting verbatim text reuse, a critical aspect of plagiarism detection. The strategic placement of suspicious text in different positions within the documents, such as at the beginning, middle, and end, mimics real-world text borrowing scenarios. This makes the corpus an ideal resource for identifying verbatim text reuse.

• Granularity levels: The availability of single sentence and single paragraph granularity levels enhances the ability to identify verbatim text reuse in both short and long content, making it suitable for diverse text analysis.
• Coherent context: The preservation of contextual coherence supports the detection of verbatim text reuse by ensuring that borrowed content blends seamlessly with the original text.

### 4.3 Author Clustering

Author clustering is another valuable application of this corpus. The diversity in writing styles and topics represented in the corpus allows for the exploration of stylometric features unique to each author. This can aid in grouping documents authored by the same writer.

• Stylometric features: The variety of writing styles present in the corpus facilitates the extraction of distinctive stylometric features, enabling accurate author clustering.
• Granularity levels: The availability of both single sentence and single paragraph granularity levels accommodates varying text lengths, further supporting authorship attribution and clustering.

### 4.4 Enhancing Academic Integrity

The study emphasizes the benchmark Corpus' critical importance in improving plagiarism detection for educational and publication purposes. It is, however, as important to investigate the larger societal implications of using specialized Natural Language Processing (NLP) models to identify plagiarism in Urdu language material.

#### 4.4.1 Preserving Academic Integrity

Implementing robust NLP models serves as a crucial mechanism for upholding academic integrity [3]. These models help to ensure the authenticity and originality of academic work by precisely recognising instances of plagiarism.

#### 4.4.2 Fostering Original Content Creation

Beyond detection, specialized NLP models actively promote a culture of creative content production. These methods allow authors to explore their unique viewpoints by discouraging and eliminating plagiarism, hence encouraging a creative environment where varied voices may thrive.

### 4.5 NLP Models and Evaluation Metrics

In the field of intrinsic plagiarism detection, utilising N-gram approaches when combined with advances in deep learning and language models provides a potential solution, especially in low-resource language contexts [4]. N-grams are used to capture linguistic patterns and context inside a document by analysing continuous sequences of 'n' objects, such as words or characters. Integrating deep learning techniques, such as BERT (Bidirectional Encoder Representations from Transformers) [5] improves the ability to comprehend intricate language structures and nuances, resulting in more effective plagiarism detection.

On the other hand, in evaluating intrinsic plagiarism detection, various metrics play a crucial role in assessing the performance of detection models [6]. Such as, accuracy provides an overall measure of correct predictions, reflecting the proportion of accurately identified instances. Precision focuses on the accuracy of positive predictions, emphasizing the relevance of identified cases. Recall, on the other hand, measures the model's ability to capture all instances of plagiarism within the dataset. The F Score combines precision and recall, offering a balanced metric for model performance. Granularity assesses the level of detail in the detection process, influencing the scope and precision of identified instances. Plagdet, WindowDiff, WindowP, WindowR, and WindowF are metrics that specifically consider the placement and alignment of detected instances, offering insights into the spatial accuracy of the model's predictions. Mean Distance provides an average measure of the distances between predicted and actual instances, offering a comprehensive evaluation of the model's overall performance in intrinsic plagiarism detection.

## 5 Future Directions

Future research could focus on enriching thematic diversity. Expanding the dataset to include a more extensive array of topics and genres would enhance its applicability across various domains, ensuring a broader representation of Urdu language writing styles. Additionally, considering the evolving landscape of linguistic expressions, periodic updates to the dataset could capture emerging trends and linguistic nuances. Furthermore, collaborations with educational institutions or online platforms could facilitate the incorporation of real-world academic essays and diverse writing samples, enriching the dataset with authentic, contemporary content.

## Ethics Statement

The authors have read and followed the ethical requirements for publication in Data in Brief and confirm that the current work does not involve human subjects, animal experiments, or any data collected from social media platforms. The authors did not need permission to use the language data from the respective news and article sites. Following are the scrapping statements of each source individually:

**Table:** 

Source of Text	Terms of Service	Copyright	Privacy	Scrapping Policies
Urdu Notes	Based on the website ToS, the web resource is allowing the data to be scrapped and distributed	The data belonging to the web resource itself	it is recommended to anonymize the data before sharing	There is no such policy
Urdu-Wikipedia	Based on the website ToS, the web resource is allowing the data to be scrapped and distributed	The data belonging to the web resource itself	it is recommended to anonymize the data before sharing	There is no such policy
Nawaiwaqt	Based on the website ToS, the web resource is allowing the data to be scrapped and distributed	The data belonging to the web resource itself	it is recommended to anonymize the data before sharing	There is no such policy
Express news	Based on the website ToS, the web resource is allowing the data to be scrapped and distributed	The data belonging to the web resource itself	it is recommended to anonymize the data before sharing	There is no such policy
Urdu Point	Based on the website ToS, the web resource is allowing the data to be scrapped and distributed	The data belonging to the web resource itself	it is recommended to anonymize the data before sharing	There is no such policy
Siasat.pk	Based on the website ToS, the web resource is allowing the data to be scrapped and distributed	The data belonging to the web resource itself	it is recommended to anonymize the data before sharing	There is no such policy
Daily Dunya	Based on the website ToS, the web resource is allowing the data to be scrapped and distributed	The data belonging to the web resource itself	it is recommended to anonymize the data before sharing	There is no such policy
Daily Waqt	Based on the website ToS, the web resource is allowing the data to be scrapped and distributed	The data belonging to the web resource itself	it is recommended to anonymize the data before sharing	There is no such policy

## Figures and Tables

**Table:** 

Source of Text	Percentage of documents in the corpus	URL	Description
Urdu Notes	20.34%	https://urdunotes.com/	Urdu Notes is a significant resource for educational content in Urdu. It offers notes, articles, and educational materials in the Urdu language, making it a valuable source for academic and learning purposes
Urdu-Wikipedia	20.21%	https://ur.wikipedia.org/	Urdu Wikipedia is a comprehensive online encyclopedia in the Urdu language. It contains a vast collection of articles covering a wide range of topics, serving as an extensive reference source
Nawaiwaqt	19.26%	https://www.nawaiwaqt.com.pk/	Nawaiwaqt is a prominent Urdu newspaper providing news, articles, and content on various subjects. It serves as a reliable source for news and information in the Urdu language
Express news	18.00%	https://www.express.com.pk/	Express News is a well-known news agency in Urdu, delivering up-to-date news, articles, and multimedia content. It plays a vital role in disseminating news in the Urdu language.
Urdu Point	10.11%	https://www.urdupoint.com	Urdu Point is a versatile platform offering news, articles, and a wide range of information in Urdu. It serves as a valuable resource for various topics in the Urdu language
Siasat.pk	6.62%	https://www.siasat.pk/	Siasat.pk is a popular online community and discussion forum in Urdu. It provides a platform for users to engage in discussions, share opinions, and access various content in the Urdu language
Daily Dunya	4.25%	https://e.dunya.com.pk/	Daily Dunya is an Urdu newspaper offering news and articles. It provides news coverage, articles, and content in the Urdu language, catering to a wide readership.
Daily Waqt	1.21%	https://www.epaper-hub.com/	Daily Waqt, accessible through ePaper-Hub, is an Urdu newspaper. It delivers news and content in the Urdu language, serving as a source of information and news

**Table:** 

Essay 01	Essay 02


## Data Availability

A Versatile Dataset for Intrinsic Plagiarism Detection, Text Reuse Analysis, and Author Clustering in Urdu (Original data) (Mendeley Data)

## Appendix B (sentence level plagiarism)

Plagiarized Document 001 (Start)

Plagiarized Document 002 (Middle)

Plagiarized Document 003 (Last)

## Appendix C (Paragraph Level Plagiarism)

Essay 01

Essay 02

Plagiarized Document 001 (Start)

Plagiarized Document 002 (Middle)

Plagiarized Document 003 (Last)
